# The landscape of non-reference SINE-VNTR-Alus in amyotrophic lateral sclerosis

**DOI:** 10.3389/ebm.2025.10600

**Published:** 2025-05-29

**Authors:** Abigail L. Pfaff, Vivien J. Bubb, John P. Quinn, Sulev Kõks

**Affiliations:** ^1^ Perron Institute for Neurological and Translational Science, Perth, WA, Australia; ^2^ Personalised Medicine Centre, Health Futures Institute, Murdoch University, Perth, WA, Australia; ^3^ Department of Pharmacology and Therapeutics, Institute of Systems, Molecular and Integrative Biology, University of Liverpool, Liverpool, United Kingdom

**Keywords:** retrotransposons, SVA, amyotrophic lateral sclerosis, neurodegeneration, genetics

## Abstract

The fatal neurodegenerative disease, amyotrophic lateral sclerosis (ALS), leads to the degeneration of motor neurons in the brain and spinal cord. Many different genetic variants are known to increase the risk of developing ALS, however much of the disease heritability is still to be identified. To identify novel genetic factors, we characterised SINE-VNTR-Alu (SVA) presence/absence variation in 4403 genomes from the New York Genome Center (NYGC) ALS consortium. SVAs are a type of retrotransposon able to mobilise in the human genome generating new insertions that can modulate gene expression and mRNA splicing and to date 33 insertions are known to cause a range of genetic diseases. In the NYGC ALS consortium sequence data 2831 non-reference genome SVAs were identified and 95% of these insertions were rare with an insertion allele frequency of less than 0.01. Association analysis of the common SVAs with ALS risk, age at onset and survival did not identify any SVAs that survived correction for multiple testing. However, there were three different rare SVA insertions in the ALS associated gene *NEK1* identified in four different individuals with ALS. The frequency of these rare insertions in *NEK1* was significantly higher in the individuals with ALS from the NYGC ALS consortium compared to the gnomAD SV non-neuro controls (p = 0.0002). This study was the first to characterise non-reference SVA presence/absence variation in a large cohort of ALS individuals identifying insertions as potential candidates involved in disease development for further investigation.

## Impact statement

This study is the first to evaluate the role of non-reference SVA variation in a large number of ALS and control genomes utilising data from the New York Genome Center ALS consortium. Common and rare SVA variants were identified in genes that could modify ALS related pathways and of note were the multiple rare insertions in the *NEK1* gene. SVAs affect genomic function through several different mechanisms and have the potential to contribute to the missing heritability of ALS.

## Introduction

The fatal neurodegenerative disease, amyotrophic lateral sclerosis, is characterised by the loss of upper and lower motor neurons resulting in muscle weakness and wasting with eventual paralysis. Respiratory failure usually occurs within 3–5 years of disease onset; however, survival times can be highly variable [1]. Both genetic and environmental factors play a role in ALS development, and it is a phenotypically and genetically heterogenous disease. The genetics of ALS is complex, with different patterns of inheritance (monogenic, oligogenic and polygenic) and gene penetrance [2]. Approximately 90% of individuals with ALS are sporadic cases and 10% have a family history, however genetic variants contribute to the development of ALS in both those with and without a family history of the disease. More than 30 genes have been associated with ALS and variants in four genes, *C9ORF72*, *SOD1*, *TARDBP* and *FUS*, have been identified in 47.7% of those with a family history and 5.2% of sporadic cases [3]. Understanding the genetic basis of ALS has provided important knowledge regarding its pathogenic mechanisms and identifying potential therapeutic targets, however there is still much to uncover regarding ALS genetics. Heritability estimates for ALS using single nucleotide variants from GWAS are much lower than those from twin studies which is often termed “missing heritability” which is estimated to be 30%–40% for ALS [4]. Part of this missing heritability may lie in large complex structural variants that have yet to be fully evaluated. Here we focus on the hominid specific retrotransposon SINE-VNTR-Alu and its potential role in ALS susceptibility.

**FIGURE 1:**
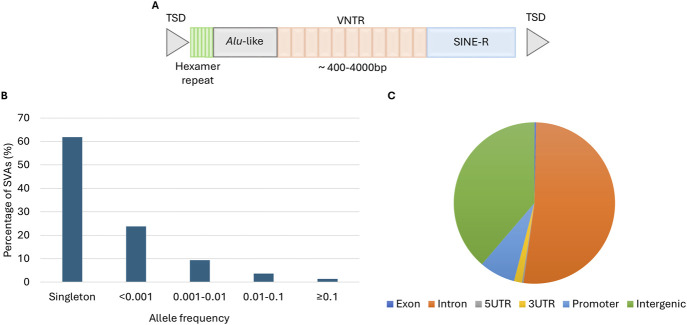
Structure, allele frequency and distribution of non-reference SVAs in the ALS consortium cohort. **(A)** A full length SVA consists of a CCCTCT-repeat variable in length, an *Alu*-like sequence, a GC-rich VNTR, a SINE region and poly A-tail flanked by target site duplications. **(B)** The percentage of SVAs with a specific insertion allele frequency in the NYGC ALS consortium. **(C)** The proportion of SVA insertions located in specific regions of the genome.

SVAs are composite elements named for their different domains, consisting of a 5′hexamer repeat, *Alu*-like sequence, GC-rich variable number tandem repeat (VNTR), SINE-region and a poly A-tail (Figure 1A) [5]. There are six subtypes of SVA (A-F) based on the sequence of their SINE region and a seventh (F1) was generated when sequence from the *MAST2* gene was incorporated through a 5′transduction event [5–7]. SVAs belong to the non-long terminal repeat (non-LTR) family of retrotransposons many of which are still actively mobilising in the human genome, both hereditary and somatic, through a ‘copy and paste’ mechanism, expanding their number and generating variation between individuals. SVAs are mobilised by the proteins encoded by the autonomous long interspersed element-1 (LINE-1) [8, 9] and using pedigree-based analysis their retrotransposition rate is estimated as 1/63 live births [10]. SVAs influence genomic function through their effects on gene expression, methylation and splicing. SVAs are known to act as expression quantitative trait loci (eQTLs) and their genotype was associated with differential gene expression in a population and tissue-specific manner [11–14]. In the brain SVAs have also been shown to be splicing, protein and methylation QTLs [12, 15] and that exonisation of SVA sequences can occur [16].

To date at least 33 SVA insertions have been identified as causing a range of genetic diseases, which includes Lynch Syndrome, Spinal Muscular Atrophy, Pompe disease, Bardet-Beidl syndrome and X-linked Dystonia Parkinsonism [17–25]. These rare insertions produce a robust phenotype and can cause disease through a range of mechanisms such as loss-of-function mutations, deletions upon their insertion into the genome and inducing aberrant splicing [22]. In addition to rare insertions with large effects, common SVA variants with small effects contribute to disease risk. One such insertion in an intron of the *CASP8* gene was associated with an increased risk of breast cancer and abnormal splicing of *CASP8* transcripts [26]. Similarly, an SVA in an intron of the *ASIP* gene that was associated with lighter skin pigmentation, an increased risk for skin cancer and changes to the splicing of the *ASIP* gene [27]. SVAs have also been associated with markers of disease progression in Parkinson’s disease [28, 29].

To investigate the role of both rare and common non-reference SVAs in ALS we utilised whole genome sequencing from 4403 individuals in the New York Genome Center (NYGC) ALS consortium dataset. We characterised the landscape of SVA variation and analysed their association with disease, age at onset and survival. The potential for their involvement in ALS is exemplified by the ALS associated gene *NEK1*.

## Materials and methods

### Genotyping non-reference SVAs using whole genome sequencing from the ALS consortium

Whole genome sequencing data in cram file format aligned to hg38 using BWA-MEM were obtained from the NYGC ALS consortium. The ALS consortium contains individuals with a range of diagnoses such as ALS spectrum MND (ALS), other MND, other neurological disorders (including Parkinson’s disease and dementias) and ALS with other neurological disorder (ALSND) as well as non-neurological controls (NNC). Non-reference SVAs were genotyped in 4403 whole genomes using Mobile Element Locator Tools (MELT version 2.2.2 in MELT-split mode) using default parameters [30]. The SVA insertions detected were filtered to keep those supported by ≥2 split reads and assess score ≥3 and that had passed the filtering criteria performed by MELT. There were 2868 non-reference polymorphic SVAs identified in the ALS consortium cohort and post filtering for Hardy-Weinberg equilibrium (p < 1 × 10^−6^ in NNC) 2831 remained. MELT does not provide information regarding SVA subtype therefore the SVAs identified here could not be analysed based on their subtype.

### Association analysis of non-reference SVAs with ALS/ALSND, age at onset and survival

Association analysis was performed on those individuals that were >90% European according to the ALS consortium metadata and those diagnosed with ALS spectrum MND or ALS spectrum MND with other neurological disorder (n = 2,656) and compared to the non-neurological controls (n = 321) (see Table 1 for demographics). Association analysis of 134 polymorphic SVAs (minor allele frequency >0.01) with ALS/ALSND was performed using logistic regression adjusted for age, sex and sequencing preparation in PLINK (v1.07) [31] and p values adjusted for multiple testing (Bonferroni correction).

**TABLE 1:** Demographics of European NYGC ALS consortium cohort in which association analysis was performed.

Demographic	NNC (n = 321)	ALS/ALSND (n = 2656)
Sex^a^		
Male	157 (48.9%)	1595 (60.1%)
Female	164 (51.1%)	1060 (39.9%)
Age^b^		
Mean (min-max)	57.5 (17–90)	59.1 (12–90)

^a^ ALS/ALSND 1 unknown.

^b^ For NNC age at collection (44 unknown) and ALS/ALSND age at symptom onset (159 unknown).

Age at onset analysis was performed using linear regression of age at onset on SVA genotype with sex, sequencing platform and site of onset as covariates and p values adjusted for multiple testing (Bonferroni correction). Survival analysis was completed using cox proportional hazards model from the “coxme” package in R with sex, sequencing platform, age at onset and site of onset as covariates and p values adjusted for multiple testing (Bonferroni correction). Individuals in the ALS consortium dataset who were still alive were censored at their last follow up.

### SVAs in ALS associated loci

To identify polymorphic SVAs from the ALS consortium data located in ALS associated loci the SVA coordinates were intersected with the coordinates of ALS associated genes. The list of ALS associated genes was generated from the amyotrophic lateral sclerosis online database (ALSoD)^1^ using those defined as a definitive ALS gene or with strong evidence for their association (Supplementary Table S1). The coordinates for the ALS genes were defined as the start of the 5′UTR to the end of the 3′UTR. In addition, location and allele frequency data for non-reference SVAs was downloaded from gnomAD SVs v4.1.0 for the non-neuro control cohort^2^ and those located in ALS associated genes were extracted.

## Results

### Distribution of non-reference SVAs in the ALS consortium cohort

A total of 2831 non-reference SVAs polymorphic for their presence/absence were identified in 4403 whole genomes from the NYGC ALS consortium cohort. The majority of the insertions were rare with 62.3% being singletons (an allele count of 1) and only 5% of insertions with an insertion allele frequency (IAF) >0.01 (Figure 1B). There was a moderate positive correlation between SVA density and gene density across the genome (*r* = 0.44, p = 3.85 × 10^−149^) and more than half of the insertions were located in a gene locus (exon or intron) (Figure 1C). This preference of SVAs in gene-rich regions is consistent with previous reports regarding SVA distribution [5, 32]. There were 10 SVA insertions (0.35%) in coding exons and 9 of those were very rare (IAF <0.0005). One exonic insertion was located in a gene, dynactin subunit 6 (*DCTN6*), that was predicted to be intolerant to loss of function variation using gene constraint metrics from gnomADv4.0. The loss of function observed/expected for *DCTN6* was 0.22 (0.1–0.57) and a threshold of <0.6 for the upper bound fraction is recommended. This particular SVA (NRSVA_8_30156399) was only found in a single individual in the NYGC consortium cohort and had been diagnosed with ALS spectrum MND.

### Association analysis of non-reference SVAs with ALS/ALSND, age at onset and survival

**FIGURE 2:**
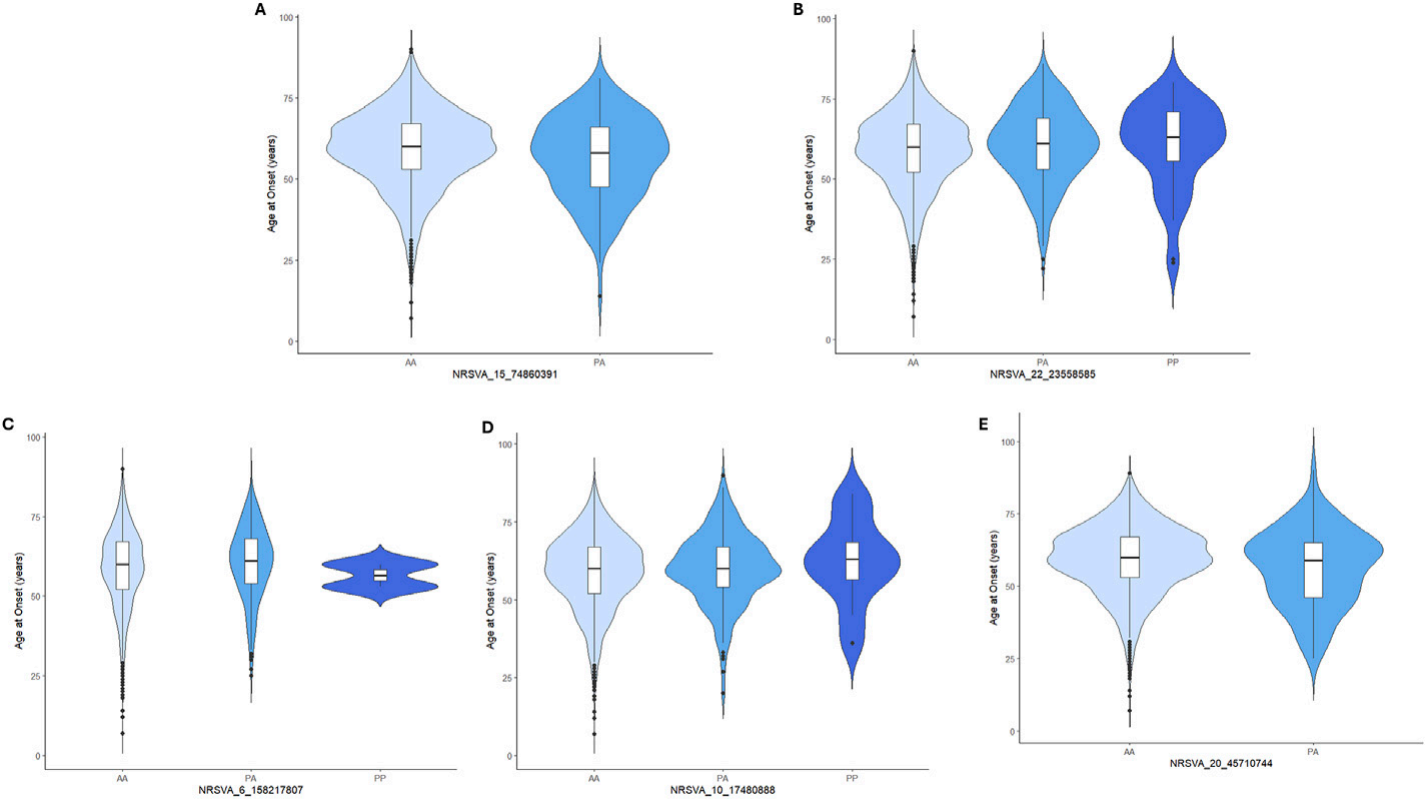
The top 5 most significant SVAs associated with age at onset prior to correction for multiple testing in the NYGC ALS consortium cohort. Each SVA and the unadjusted p values are reported in the figure as follows: **(A)** NRSVA_15_74860391 p = 0.001, **(B)** NRSVA_22_23558585 p = 0.005, **(C)** NRSVA_6_158217807 p = 0.02, **(D)** NRSVA_10_17480888 p = 0.02 and **(E)** NRSVA_20_45710744 p = 0.03.

**FIGURE 3:**
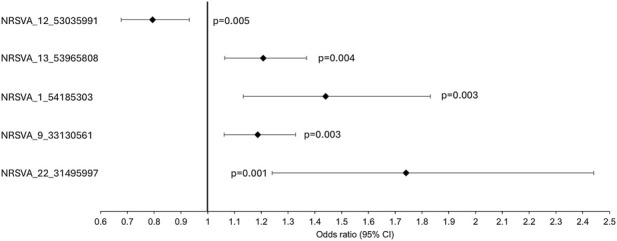
The top 5 most significant SVAs associated with survival prior to correction for multiple testing in the NYGC ALS consortium cohort. The black diamonds represent the hazard ratio and black lines the 95% confidence intervals and unadjusted p values are reported.

Association analysis of 134 SVAs (MAF >0.01) with ALS/ALSND, age at onset and survival was performed on the European subset of the NYGC ALS consortium (see Table 1 for demographics) and no SVAs survived correction for multiple testing (Supplementary Tables S2–S4). There were 10 SVAs that were significantly associated with disease prior to correction and the top 5 are shown in Table 2. In the age at onset analysis the top 5 SVAs significant prior to correction are shown in Figure 2. There were 14 SVAs significantly associated with survival prior to correction with the top 5 shown in Figure 3.

**TABLE 2:** Top 5 significant non-reference SVAs significantly associated with ALS prior to correction.

SVA ID	Minor allele	Minor	MAF		Unadj p value	Bonferroni p value
		NNC	ALS/ALSND	OR (95% CI)		
NRSVA_12_104417657	P	0.086	0.064	0.65 (0.47–0.91)	0.012	1
NRSVA_17_16359803	P	0.031	0.019	0.52 (0.31–0.87)	0.013	1
NRSVA_5_32283375	P	0.037	0.019	0.56 (0.34–0.92)	0.023	1
NRSVA_14_35375522	P	0.078	0.11	1.45 (1.04–2.03)	0.029	1
NRSVA_22_31495997	P	0.0016	0.016	9.03 (1.25–65.1)	0.029	1

Of those SVAs significant prior to correction three were significant in two of the analyses performed and may warrant further investigation in future studies. NRSVA_13_53965808 located in an intron of the long non-coding RNA LINC00458 was associated with ALS/ALSND prior to correction (unadjp = 0.049, OR = 1.3 (1.001–1.759)) and with reduced survival time (unadjp = 0.004, HR = 1.21 (1.06–1.37)). Individuals homozygous absent (AA) for NRSVA_13_53965808 had a disease duration of 43.0 months whereas this was reduced to 38.8 months for heterozygous individuals (PA) and 20 months for homozygous present (PP). NRSVA_15_74860391 located in an intron of the *SCAMP2* gene was associated with age at onset prior to correction (unadjp = 0.001) with a mean age at onset of 59.2 years for those with AA genotype and 56.8 years for those with PA genotype. NRSVA_15_74860391 was also associated with reduced survival time prior to correction (unadjp = 0.034, HR = 1.31 (1.02–1.70)) and AA individuals had a mean survival time of 42.0 months compared to PA individuals with 35.2 months. The third SVA to be significant in the two of the analyses performed was NRSVA_22_31495997 located in the 5′UTR of a transcript of the *EIF4ENIF1* gene, the promoter and 150bp upstream of the *SFI1* gene and in an intron of the *DRG1* gene. NRSVA_22_31495997 was associated with ALS/ALSND prior to correction (unadjp = 0.029, OR = 9.03 (1.25–65.1)) and reduced survival time (unadjp = 0.001, HR = 1.74 (1.24–2.44)) with a mean disease duration of 42.6 months for AA individuals and 31.3 months for PA individuals.

### Rare SVA insertions are located in ALS associated genes

**FIGURE 4:**
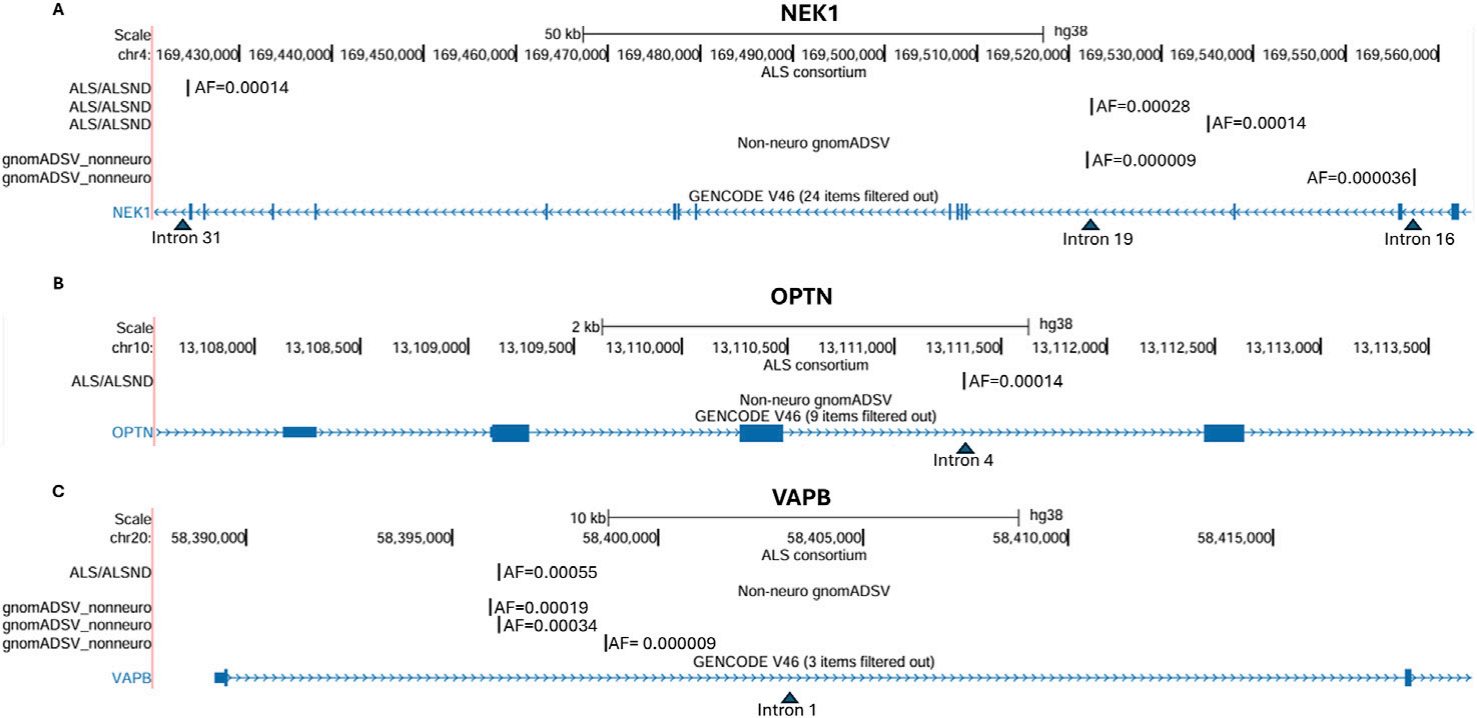
Rare SVA insertions located in ALS associated genes. **(A)** The location of three SVA insertions in the *NEK1* gene identified in four individuals with ALS/ALSND in the ALS consortium and two SVA insertions identified in five individuals in the non-neuro controls from gnomAD SV. **(B)** The location of an SVA insertion in the *OPTN* gene identified in one individual with ALS/ALSND in the ALS consortium. **(C)** The location of an SVA insertion in the *VAPB* gene identified in four individuals with ALS/ALSND in the ALS consortium and three SVA insertions identified in 60 individuals in the non-neuro controls from gnomAD SV.

Our association analysis was performed for those SVAs with a MAF >0.01, however this is only a small proportion of the insertions identified (5%) therefore we assessed the location of all SVAs in relation to ALS associated genes. A list of 21 genes that were classified as definitive ALS genes or with strong evidence for their association was taken from the amyotrophic lateral sclerosis online database (ALSoD).^1^ From the 2831 SVAs genotyped 5 rare SVAs were in 3 ALS associated genes (*NEK1*, *OPTN* and *VAPB*) (Figure 4; Table 3) and all were identified in individuals diagnosed with ALS/ALSND. However, the number of ALS/ALSND was much higher than the number of controls in the ALS consortium cohort analysed (3603 vs. 425), therefore we assessed the presence SVAs in the 3 ALS associated genes in the non-neuro controls from gnomAD SVs v4.1.0 (55390 individuals).

**TABLE 3:** Location and allele frequency of SVAs in ALS associated genes.

ALS gene	NYGC ALS consortium ALS/ALSND					gnomAD SV non-neuro controls				
	Chr	Start	Location	SVA IAF	Combined IAF	Chr	Start	Location	SVA IAF	Combined IAF
NEK1	4	169522361	Intron 19	0.00028	0.00056	4	169521879	Intron19	0.000009	0.000045
	4	169535006	Intron19	0.00014						
	4	169424340	Intron 31	0.00014		4	169557353	Intron 16	0.000036	
OPTN	10	13111322	Intron 4	0.00014	0.00014					
VAPB	20	58396121	Intron 1	0.00056	0.00056	20	58395922	Intron 1	0.00019	0.00054
						20	58396121	Intron 1	0.00034	
						20	58398736	Intron 1	0.000009	

In *NEK1* three different intronic SVA insertions in four individuals diagnosed with ALS/ALSND were identified and in the gnomAD SV non-neuro controls two intronic SVA insertions were identified in five individuals (Figure 4A). The combined allele frequency of those SVAs in the ALS/ALSND group was 0.00056 which was significantly higher than the combined allele frequency of 0.000045 in the gnomAD SV non-neuro controls using Fisher’s exact test (p = 0.0002, OR = 12.6 (3.4–47.0)) (Table 3). An intronic SVA was detected in a single individual diagnosed with ALS/ALSND in the *OPTN* gene and there were no SVA insertions located in the gene in the gnomAD SV group (p = 0.018, OR = 47.2 (1.9-INF)) (Figure 4B; Table 3). Finally, an SVA was identified in four individuals with ALS/ALSND and three different SVAs in 60 individuals from the gnomAD SV non-neuro controls and all were in intron 1 of the *VAPB* gene (Figure 4C). The combined allele frequencies of the SVAs in the *VAPB* gene were very similar between the two cohorts (p = 0.92, OR = 1.05 (0.35–2.89)) (Table 3). There were fixed SVAs present in the reference genome in intron 12 of *NEK1* and intron 1 of the *VAPB* and no SVAs in the reference sequence of *OPTN*.

## Discussion

We performed genome-wide analysis of non-reference SVA insertions in 4403 genomes from the NYGC ALS consortium to investigate their role in ALS and to determine if these elements could fill in part of the missing heritability of the disease. We identified 2831 SVAs and evaluated both common and rare insertions identifying potential candidates for further investigation in ALS development.

To evaluate the role of common SVA insertions (IAF >0.01) in disease risk, age at onset and survival association analysis was performed and of the 134 SVAs (IAF >0.01) analysed there were no insertions significant after correction for multiple testing. Although no significant associations were detected in this cohort future studies with a larger number of samples may identify disease associated elements. Of particular interest would be those three SVA insertions identified as significant prior to correction in two out of the three analyses and could affect ALS related pathways. Two of these SVAs were located in introns; one SVA in a long non coding RNA (LINC00458) whose sequence is in part derived from a human endogenous retrovirus and is involved in promoting pluripotency [33, 34]. The second intronic SVA was in the *SCAMP2* a gene that encodes a secretory carrier membrane protein. Defects in vesicular transport have been associated with the pathogenesis of ALS and several genes associated with ALS, such as *VAPB, C9ORF72* and *SOD1*, are involved in this pathway [35]. The third SVA was located in the 5′UTR of a transcript of the *EIF4ENIF1* gene encoding a nucleocytoplasmic shuttle protein for the translation initiation factor eIF4E and in the promoter of the *SFI1* gene encoding a centrin binding protein.

The majority of the SVA insertions identified in the NYGC ALS consortium were rare; 95% had an IAF <0.01 and 62% were only found in a single individual. Low allele frequencies and a large proportion of singletons have been reported in population studies of SVAs, which include data from the Human Genome Diversity Project, Simons Genome Diversity Project, 1000 Genomes Project and gnomAD-SV [36, 37], as these retrotransposons are biologically young and still active in the human genome. The rare variants identified in the ALS consortium cohort were prioritised based on either causing a loss of function (insertions in coding exons) or located in ALS associated genes. Of the 10 genes containing an exonic SVA only 1 showed an intolerance to loss of function variation according to the gnomAD database [38]. This SVA had inserted into the first coding exon of the *DCTN6* gene and is likely to introduce a premature stop codon within the gene transcript, which is a mechanism reported for several disease causing SVAs [22]. DCTN6 forms part of the dynactin protein complex and is involved in dynein mediated retrograde transport [39, 40]. The *DCTN6* gene has not previously been associated with ALS, however mutations in another member of the dynactin-complex, *DCTN1*, have been identified in individuals with ALS and defects in axonal transport are associated with ALS development [41–43]. Identification of other individuals with ALS who have variants in *DCTN6,* and their functional relevance will be required to determine what role this SVA may be playing in the disease.

We then evaluated the rare insertions in ALS associated genes identifying 5 SVAs in 3 genes (*NEK1*, *OPTN* and *VAPB*) in 9 individuals with ALS/ALSND. These insertions were not detected in the NNC cohort of the NYGC ALS consortium. However, the number of NNCs is 8.5 times lower than the number of ALS/ALSND individuals, which is one of the limitations of this study due to the imbalance of cases and controls. Therefore, we utilised the non-neuro controls cohort from the gnomAD SVs v4.1.0 database to determine the allele frequency of these SVAs in a much larger control cohort, 15.4x larger than the ALS/ALSND NYGC cohort analysed. Supplementing controls from gnomAD can improve statistical power [44] and provides an important reference database especially when evaluating rare variants. Using the gnomAD SV data we were able to show there was no significant difference in the allele frequency of the SVAs in the *VAPB* gene and unlikely to be involved in disease. The SVA in an intron of the *OPTN* gene of a single individual with ALS was not present in the gnomAD SV non-neuro controls. Different types of mutations have been identified in the *OPTN* gene, including missense, frameshift and deletions, and show both autosomal dominant and recessive patterns of inheritance in ALS [45]. The combined allele frequency of the SVAs in the *NEK1* gene was significantly higher in the ALS/ALSND groups than the gnomAD SV non-neuro controls (0.00056 compared to 0.000045). NEK1 is involved in several cellular process, such as the DNA damage response and cell cycle regulation, and both rare and common variants of the gene have been associated with ALS risk [46].

The SVAs in *OPTN* and *NEK1* were intronic and although SVAs are frequently found in introns half of the disease causing SVA insertions identified to date are intronic and have significant effects on splicing patterns. SVAs contain multiple cryptic splice sites within their sequence therefore intronic SVAs can be exonised and included in the gene transcript, often introducing premature stop codons [16, 47]. SVAs have also been shown to activate existing cryptic splice sites located in the intron in which they have inserted inducing aberrant splicing [22]. Intronic SVAs have the potential to affect the function of the gene in which they have inserted, and further work would be required to determine the effects of the SVAs that were identified in the ALS associated genes *OPTN* and *NEK1*.

Our study was the first to characterise non-reference SVA variation in a large cohort of ALS individuals identifying insertions as potential candidates involved in disease development. We have identified SVA variants that would be predicted to modify signalling pathways involved in ALS. SVAs can influence the transcriptome and affect cellular function and therefore are an important type of genetic variation to be evaluated when investigating the missing heritability of disease.

## Data Availability

The WGS sequencing analysed in this study from the ALS consortium were obtained upon application to the New York Genome Center and data requests can be made by completing a genetic data request form at ALSData@nygenome.org. https://www.nygenome.org/science-technology/collaborative-research.

## Footnotes

1
https://alsod.ac.uk/

2
https://gnomad.broadinstitute.org/downloads
